# A Systematic Review of Patients’ Experiences in Communicating with Primary Care Physicians: Intercultural Encounters and a Balance between Vulnerability and Integrity

**DOI:** 10.1371/journal.pone.0139577

**Published:** 2015-10-06

**Authors:** Rhea Rocque, Yvan Leanza

**Affiliations:** School of Psychology, Laval University, Quebec City, Quebec, Canada; University of Louisville School of Medicine, UNITED STATES

## Abstract

Communication difficulties persist between patients and physicians. In order to improve care, patients’ experiences of this communication must be understood. The main objective of this study is to synthesize qualitative studies exploring patients’ experiences in communicating with a primary care physician. A secondary objective is to explore specific factors pertaining to ethnic minority or majority patients and their influence on patients’ experiences of communication. Pertinent health and social sciences electronic databases were searched systematically (PubMed, Cinahl, PsychNet, and IBSS). Fifty-seven articles were included in the review on the basis of being qualitative studies targeting patients’ experiences of communication with a primary care physician. The meta-ethnography method for qualitative studies was used to interpret data and the COREQ checklist was used to evaluate the quality of included studies. Three concepts emerged from analyses: negative experiences, positive experiences, and outcomes of communication. Negative experiences related to being treated with disrespect, experiencing pressure due to time constraints, and feeling helpless due to the dominance of biomedical culture in the medical encounter. Positive experiences are attributed to certain relational skills, technical skills, as well as certain approaches to care privileged by the physician. Outcomes of communication depend on patients’ evaluation of the consultation. Four categories of specific factors exerted mainly a negative influence on consultations for ethnic minorities: language barriers, discrimination, differing values, and acculturation. Ethnic majorities also raised specific factors influencing their experience: differing values and discrimination. Findings of this review are limited by the fact that more than half of the studies did not explore cultural aspects relating to this experience. Future research should address these aspects in more detail. In conclusion, all patients seemed to face additional cultural challenges. Findings provide a foundation for the development of tailored interventions to patients’ preferences, thus ensuring more satisfactory experiences. Health care providers should be sensitive to specific factors (cultural and micro-cultural) during all medical encounters.

## Introduction

Physician-patient communication is a central element in primary care consultations and has been described as having specific purposes. For instance, adequate communication fosters the development of a satisfactory relationship, allows patient and physician to engage in proper information exchange, decide on a treatment plan, and ensure adherence to treatment [1, 2]. Moreover, a favorable communication has been linked to many positive outcomes, such as patient satisfaction with care, higher quality of care and of physician-patient relationship, adherence to treatment, and to better objective physical outcomes [3–8].

Although benefits associated to favorable physician-patient communication are well documented, communication difficulties in medical encounters persist. Such difficulties can engender serious consequences. For instance, inadequate communication has been linked to patient dissatisfaction with care, incomprehension of treatment plan, non-adherence to treatment, lower quality of care and of physician-patient relationship, overutilization or underutilization of resources, and medical errors [4, 7, 9, 10].

Communication difficulties are unfortunately particularly relevant with ethnic minority patients (EMPs). For instance, during consultations with EMPs, physicians behave less affectively, more misunderstandings occur, patients report lower satisfaction with care and with communication, and studies document poorer adherence to treatment [6, 11].

Considering culture’s important role in the organisation and interpretation of one’s experiences, its role in the interpretation and transmission of messages should not be neglected. Indeed, communication is intrinsically cultural. On top of transmitting objective information, communication also conveys emotions, implicit content, and implicit meanings, all of which are derived from one’s cultural systems. In light of this information, communication difficulties between EMPs and physicians may be explained by differences in cultural backgrounds of both interlocutors.

However, a great majority of studies exploring physician-patient communication consider culture as the equivalent of ethnicity and sometimes race. Although ethnicity and race have been defined in many ways, these studies equate these terms to “*the place of origin where the group’s culture emerged as a distinct entity*” [12, p10] or to the US census categories that emerged in the late 19th and early 20th centuries which were then criticized for being openly racist “scientific” categories [13]. Although some biomedical papers offer a definition of race and ethnicity, most papers using such terms to categorize human beings do not define them or do not explain differences they find between these groups [14]. This absence of definition limits the understanding of complex sociocultural aspects at play in communication.

Intercultural studies aiming to better understand communication between patients and physicians would gain a lot in considering a broader definition of both culture and ethnicity. Regarding culture, Spencer-Oatey and Franklin write that “*there are many different types of social groups*, *and where members of any group share patterns of regularity in some way […]*, *they can be regarded as belonging to a cultural group*” [15, p40]. According to this definition, individuals may belong simultaneously to many cultural groups. Indeed, all communication takes place not only within a large cultural context but also within multiple micro-cultural contexts [16]. Clanet defines culture as “*a set of systems of meanings that belong to a group or a sub-group*, *meanings that appear as values and give birth to rules and norms that the group will conserve and transmit*, *meanings through which the group will differentiate itself from neighbouring groups*” (free translation) [17, p15]. This definition illustrates the central role culture plays in helping individuals make sense of their universe and experiences, as well as helping individuals establish norms that will in turn guide their daily behaviours. Ethnicity, in turn, refers to the process in which individuals engage in when manipulating symbols and meanings in order to put forth a certain aspect of their identity in a given context, thus allowing them to cross cultural or micro-cultural boundaries and play with their different identities [18].

Culture, micro-cultures and ethnicity thus play important roles in the transmission and interpretation of messages. Applied to medical encounters, this conceptualisation suggests that all medical encounters are intercultural from the outset, since being a physician involves having been socialised in a specific micro-culture; biomedicine. To this regard, Beagan et al. [19] show that medical socialisation neutralizes sociocultural differences among medical students. Consequently, medical students tend to overlook and neglect sociocultural influences in medical encounters.

However, cultural and micro-cultural influences should not be disregarded when exploring physician-patient communication, since ignoring such influences can contribute to maintaining health disparities and discrimination in healthcare and beyond [20, 21]. Indeed, the lack of sensitivity to cultural aspects can lead to perceptions of discrimination; a subjective experience that has been demonstrated to engender adverse effects on health in the long term, thus perpetuating health disparities [22].

### Previous Literature Reviews

Results from previous reviews provide valuable insight with regard to which behaviours foster satisfaction among patients, patients’ expectations and desires with regard to communication, and to potential specific factors that may impede on communication with EMPs [6, 9, 23]. However, they present some limitations that restrict our understanding of communication difficulties. First, previous reviews neglected to consider patients’ lived experiences either by including only observational studies seeking to establish associations between physicians’ behaviours and various outcomes (e.g. patient satisfaction) [6, 9] or by focusing only on patients’ expectations of what a consultation should be like and on patients’ preferences and desires regarding physicians’ behaviours [23]. Meanwhile, authors emphasize the need to consider the patient’s lived experience in order to tailor interventions and communication [24–26]. Second, quantitative methods predominate in previous reviews. Although quantitative methods allow establishing associations between variables, qualitative studies should also be examined since they allow a profound and detailed understanding of patients’ experiences. Third, cultural aspects at play in communication are not explored frequently in previous reviews. Besides, studies included in reviews that explore cultural aspects most often equate culture with ethnicity. Neglecting culture or reducing it to “race” or “ethnic” categories tends to biologize sociocultural isssues [14] and to maintain health disparities and discrimination in healthcare and beyond [22]. To the best of our knowledge, no previous review has explored, from the patient’s perspective, potential broader cultural influences on patients’ experiences. Considering culture’s central role in the transmission and interpretation of messages, its influence should be explored.

### Study Purpose

Qualitative studies targeting patients’ experiences in communication have been conducted but these studies remain scattered and must be synthesized in order to gain a global understanding of patients’ experiences. A global understanding of experiences of a culturally diverse patient sample could help shed light on both universalities cutting across cultures and particularities relating to incarnating the patient role.

The current systematic review has three objectives. First, it aims to explore patients’ experiences in communicating with a primary care physician (PCP). Second, it intends to investigate specific factors pertaining to EMPs and their influence on EMPs’ experiences in medical encounters. The third objective is exploratory in nature and consists of investigating specific factors related to micro-cultural belonging which influence ethnic majority patients’ experiences in medical encounters. More precisely, three research questions are to be answered:

1. What are patients’ experiences in communicating with a primary care physician?
2. What specific factors influence ethnic minority patients’ experiences of communication?
3. What specific factors influence ethnic majority patients’ experiences of communication?

## Method

### Sampling

**Fig 1:**
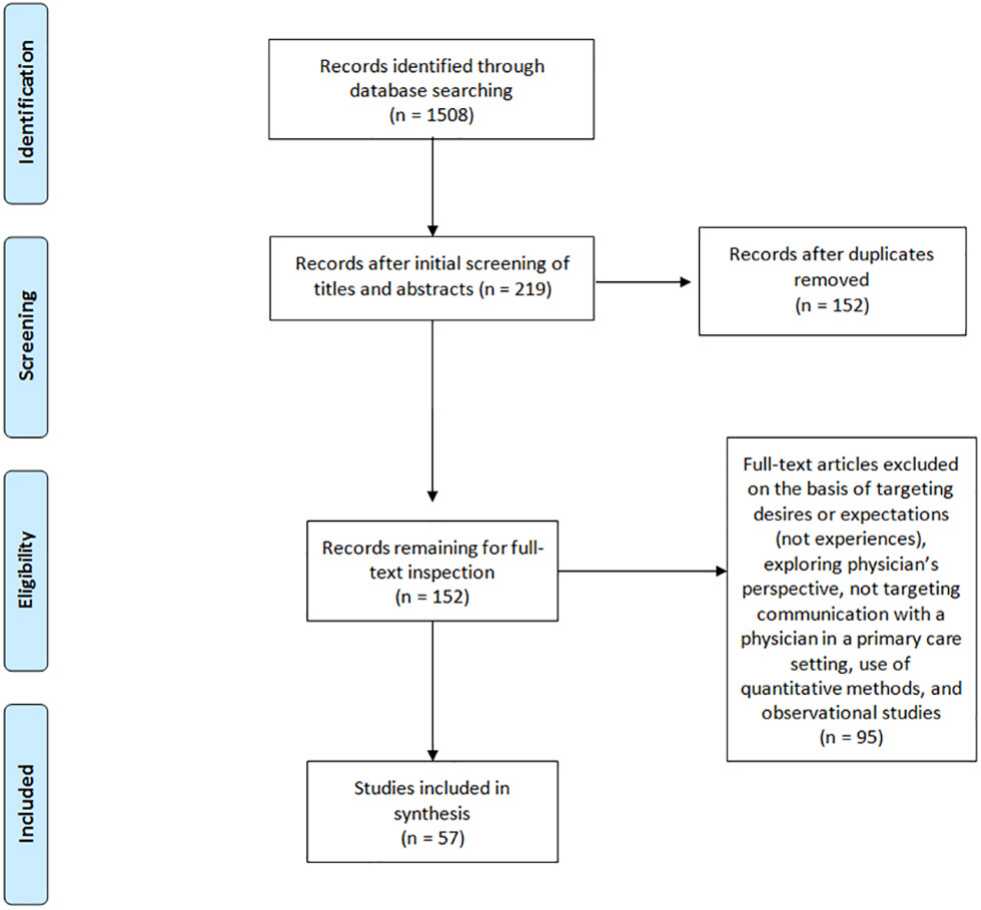
Flowchart for the three step article selection process.

An electronic search of relevant databases was conducted for qualitative studies targeting patients’ experiences in communicating with a PCP. No date criterion was set and only articles published in French or English were eligible. Two researchers entered the same key-words in four databases (PubMed, Cinahl, PsycNET, and IBSS) and independently selected articles. Keywords were “physician-patient relations” and “health communication” and “qualitative”. See Fig 1 for an overview of the selection process. The initial search conducted in August 2015 yielded 1508 results (1029 Pubmed, 397 Cinahl, 26 IBSS, 56 PsycNET). Articles were first selected by reading the title, next, by examining the abstract, and last, by reading the full text. When titles did not provide enough information to classify articles, abstracts or full text were inspected. Articles were excluded if they did not target patients’ experiences of face-to-face communication with a PCP using a qualitative design. The first selection, according to titles and abstracts, yielded a total of 219 articles. During this first selection step, the goal was not to reach perfect inter-rater agreement, but to include the largest amount of studies. After deletion of duplicates, 152 articles remained for full text inspections. During this step, 95 articles were excluded on the basis of targeting patients’ desires and expectations (11), targeting physicians’ perspective (2), not targeting communication in primary care settings (50), not targeting face-to-face communication (22) or targeting communication with the health care system in general (4), and for being quantitative or mixed-methods studies focusing mostly on quantitative results (4) or observational studies (2). Following full text inspection, 57 articles remained.

### Study Design & Data Analysis

Noblit and Hare’s [27] meta-ethnography method was utilized to synthesize qualitative studies. In short, their method involves an in-depth reading of selected articles, followed by a synthesis of studies. To synthesize studies, common concepts across studies must be identified and the relationship between these concepts must be described.

A previous review of qualitative studies in the field of healthcare communication has successfully applied this method to review literature relating to working with interpreters [28]. Furthermore, Britten et al. undertook a worked example and conclude that their meta-ethnography (and thus potentially future meta-ethnographies) “*offers evidence that qualitative synthesis is possible and may be able to provide clearer and more succinct findings for practitioners and policymakers than individual studies or narrative reviews*” [29, p215].

The matrix method was employed to organize and analyze the data [30]. Garrard [30] recommends the use of a spreadsheet software which allows an easy comparison of studies. For this review, each article occupied a row and columns were organized to document authors, year of publication, journal, country, sample size and description, study objectives, method, a summary of findings, and syntheses.

More concretely, each study was first summarized under one column named ‘Summary of findings’. Summaries were then read in-depth, in order to inductively identify common concepts. For each emerging concept, a new matrix was created and summaries of studies were incorporated in the matrix. A first synthesis occurred in order to describe how each concept evolved from one study to the next (see Results). Next, a “line-of-argument synthesis”, as described by Noblit & Hare [27], was undertaken in order to explain how each concept related to one another from one study to the next. This second type of synthesis calls for interpretation of relations between concepts with the aim of producing an explanation of the phenomenon under study (see Discussion).

### Quality Evaluation

Tong et al. [31] developed a checklist to guide readers in the quality evaluation of qualitative articles. The checklist is divided in three domains which should be discussed in order to allow readers to evaluate quality: research team and reflexivity, study design, and analyses and findings. Fifty-two of the 57 articles discussed aspects from all three domains and were evaluated as possessing a good quality. Five articles neglected to present information relating to the first domain. They were nevertheless judged as good quality since they were shorter papers and might simply have faced space restrictions (see S1 Table for details).

## Results

### Description of Studies

**Fig 2:**
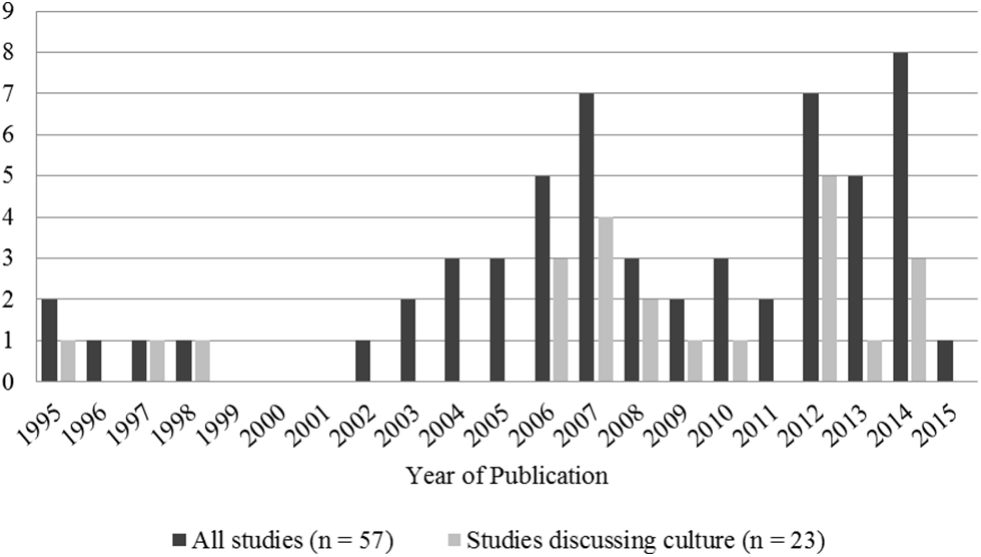
Number of publications per year and number of publications per year discussing culture.

**Fig 3:**
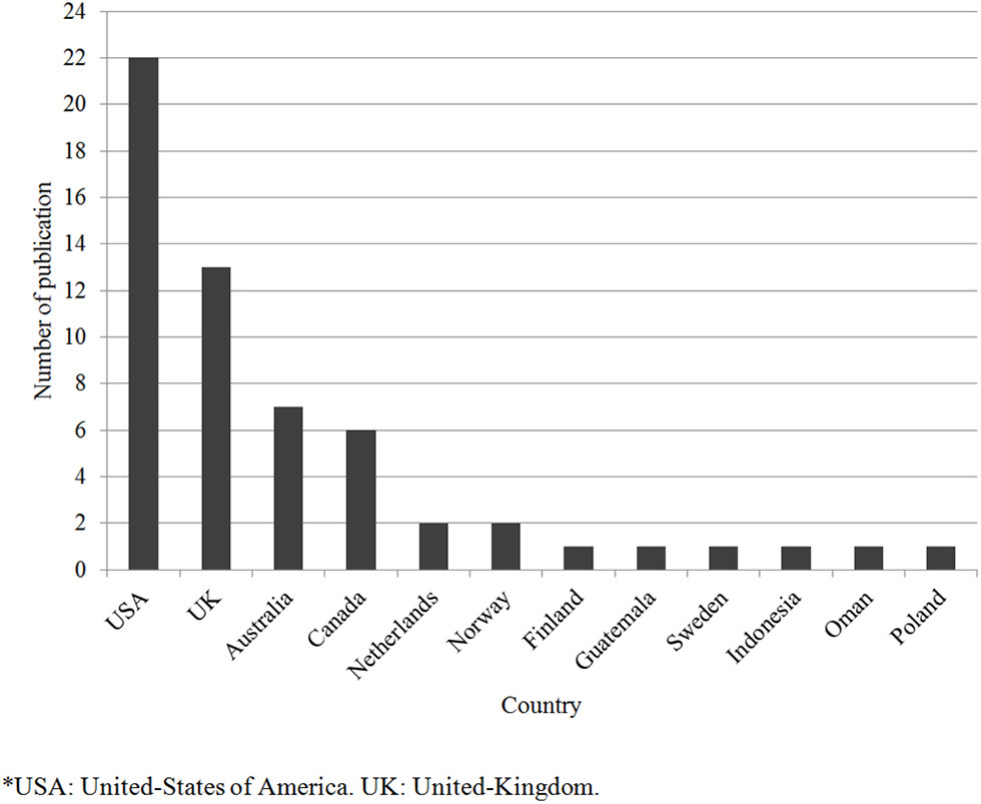
Number of publications by country.

**Fig 4:**
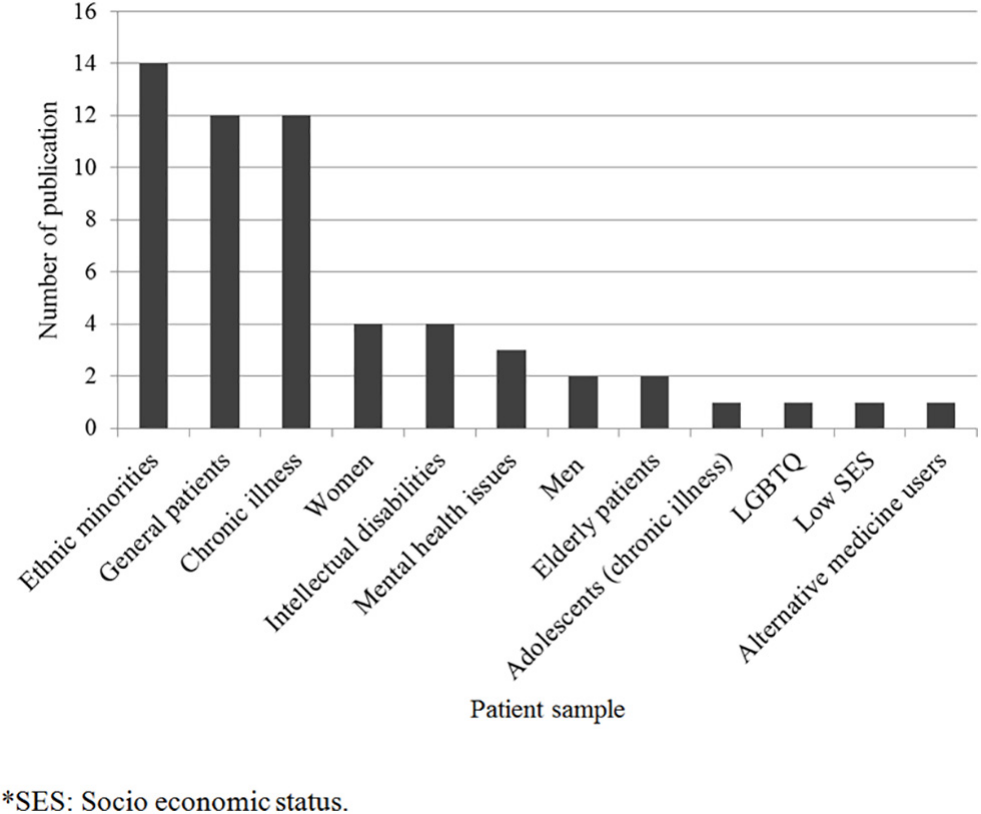
Number of publications according to patient sample.

Publication dates varied between 1995 and 2015 (Fig 2). The majority of studies were conducted in North America (n = 28) and in Europe (n = 20; Fig 3). A total of 1872 patients took part in the studies included in this review. As can be seen in Fig 4, patients belonging to many cultural and micro-cultural groups were included in this review. Twelve of the studies did not specify a cultural group (referred to as the general patient population) and 14 studies targeted specifically EMPs. See Table 1 for an overview of studies.

**Table 1:** Overview of studies targeting patients’ experiences in communicating with a primary care physician (n = 57): authors, year and country of publication, aim, sample, method, and attention to cultural aspects.

Author (Year)	Country	Aim	Sample	Method & Analyses	Culture
Bowes & Domokos (1995) [32]	UK^a^	Explore South Asian women's experiences and use of health services	20 Pakistani Muslim women (17 born abroad)	UII^b^ Thematic	√^c^
Punamaki & Kokko (1995) **[33]**	Finland	Describe and analyze patients' consultation experiences	127 patients or parents of patients (60F, 67M) (ENR^d^)	SSI^e^ Thematic	
Johansson, Hamberg, Lindgren & Westman (1996) **[34]**	Sweden	Explore female patients’ experiences in consultation	20 Swedish female with musculoskeletal disorders	SSI Grounded Theory	
**Thom, Campbell & Alto (1997) [35]**	USA^f^	Identify physician behaviours that foster trust	29 patients (1 FG^g^ Hispanics, 1FG African Americans, 2 FG not reported but inferred as White Americans) (20F, 9M)	4 FGs Grounded Theory	√
**Rodriguez, Bauer, Flores-Ortiz, Szkupinzki-Quiroga (1998) [36]**	USA	Identify provider related factors that may affect patient-provider communication about abuse for immigrant women	14 Hispanic and 14 Asian women (n = 28)	4 FG: 2 Hispanic, 2 Asian Thematic	√
**Pollock & Grime (2002) [** 37 **]**	UK	Compare physicians’ and patients’ perspective of time constraints	60 adults diagnosed with depression (ENR)	II^h^ Thematic	
**Beresford & Sloper (2003) [** 38 **]**	UK	Identify factors that hinder or facilitate communication	63 adolescents with chronic physical illness (36F, 27M) (ENR)	SSI Thematic	
Gask, Rogers, Oliver, May & Roland (2003) **[** 39]	UK	Explore experiences of care that depressed patients receive from their physician	27 patients with mild depression (19F, 8M) (ENR)	SSI Thematic	
**Walter, Emery, Rogers & Britten (2004) [** 40 **]**	UK	Examine women's perspectives of optimal risk communication and decision making	34 native English-speaking women in menopause, 6 non-native English-speakers (n = 40)	FG & 4 SSI Thematic	
**Ziviani, Lennox, Allison, Lyons & Del Mar (2004) [** 41 **]**	Australia	Identify critical factors and obstacles to effective health communication	3 adults with intellectual disabilities (2F, 1M) (ENR)	SSI Thematic	
Dubé, Fuller, Rosen, Fagan, O'Donnell (2004) **[** 42]	USA	Examine communication issues important to men on cancer screening topics	29 White, 8 Black, 5 Hispanic, 11 biracial, other and unspecified men (n = 53)	8 FGs Thematic	
**Ellis & Campbell (2005) [** 43 **]**	USA	Explore the importance of concordant spiritual belief systems in patient-physician interactions	1 Jewish, 6 Christian, 2 Agnostic, 1 Buddhist (6F, 4M) (n = 10)	SSI Thematic	
**O'Day, Killeen, Sutton & Lezzoni (2005) [** 44 **]**	USA	Explore perspective of psychiatrically ill patients receiving primary care	11 Caucasian, 5 other with chronic psychiatric disorders (8F, 8M) (n = 16)	FG Thematic	
**Sankar & Jones (2005) [** 45 **]**	USA	Understand patient's perspective on information disclosure	26 African American, 8 Asian, 5 Hispanic, 39 White, 4 Other women (n = 85)	II Thematic	
**Ali, Atkin & Neal (2006) [** 25 **]**	UK	Understand the ways in which White and South Asian patients communicate with White physicians	7 White, 18 South Asian (21F, 4M) (n = 25)	Video recall and SSI Thematic	√
**Cant & Taket (2006) [** 46 **]**	UK	Explore lesbians’ and gays’ experiences of primary care	18 White, 5 Black and other ethnic minority (13F, 10M) (n = 23)	FG Grounded Theory	√
Moffat, Cleland, van der Molen & Price (2006) **[** 47]	UK	Explore patients' experiences of consultations regarding asthma	14 patients with severe asthma (8F, 6M) (ENR)	SSI Grounded Theory	
**Towle, Godolphin & Alexander (2006) [** 48 **]**	Canada	Understand the complexity of physician-patient communication in Aboriginal communities	26 Aboriginal patients (22F, 4M)	8 SSI & 3 FGs Thematic	√
**Vickers, Jolly & Greenfield (2006) [** 49 **]**	UK	Explore knowledge, attitudes, and behaviours about herbal medicine and use disclosure	18 White British female herbal medicine users	II Thematic	
Abdulhadi, Shafaee, Freudenthal, Östenson & Wahlström (2007) **[** 50]	Oman	Explore views of type 2 diabetic patients regarding medical encounter	27 Omani type 2 diabetes patients (14F, 13M)	4 FG Thematic	√
Borgsteede, Deliens, Graafland-Riedstra, Francke, van der Wal & Willems (2007) **[** 51]	Netherlands	Explore patients’ experiences of communicating about euthanasia	12 patients with short life expectancy (ENR)	SSI Thematic	
Fagerli, Lien & Wandel (2007) **[** 52]	Norway	Explore patients’ experiences of medical encounters	15 Pakistani born type 2 diabetes patients (11F, 4M)	SSI Phenomenology	√
Kokanovic & Manderson (2007) **[** 53]	Australia	Describe the way patients in an Australian setting are told of diabetes	8 Chinese, 8 Indian, 8 Pacific Island, 8 Greek type 2 diabetes patients (16F, 16M) (n = 32)	SSI Thematic	√
Lowe, Griffiths & Sidhu (2007) **[** 54]	UK	Explore attitudes and experiences of South Asian women towards contraceptive service provision	19 Pakistani women (2 born in UK, 17 born abroad)	SSI Grounded Theory	√
Mercer, Cawston & Bikker (2007) **[** 55]	UK	Explore patients' views on determinants of quality of consultations in an economically deprived community	72 White Caucasian low SES^i^ patients (44F, 28M)	11 FGs Grounded Theory	
Oliffe & Thorne (2007) **[** 56]	Canada, Australia	Explore male patients’ experiences of interactions with male physicians about prostate cancer	33 Australian, 19 Canadian men with prostate cancer (n = 52)	SSI Thematic	
Julliard, Vivard, Delgado, Cruz, Kabak & Sabers (2008) **[** 57 **]**	USA	Clarify which conditions reinforce nondisclosure of health information in clinical encounters between Latina patients and their physicians	28 Hispanic women (8 born in US, 20 born in South or Central America)	SSI Grounded Theory	√
Nguyen, Barg, Armstrong, Holmes & Hornik (2008) **[** 58]	USA	Examine elements of physician-patient cancer communication from the viewpoint of older Vietnamese immigrants	20 Vietnamese immigrants	SSI Grounded Theory	√
Smith, Braunack-Mayer, Wittert & Warin (2008) **[** 59]	Australia	Examine men’s experiences of communicating with physicians in order to describe qualities and styles of communication that men prefer	30 Australian, 6 British men (n = 36)	SSI Thematic	
Shelley, Sussman, Williams, Segal & Crabtree (2009) **[** 60]	USA	Compare patients’ and physicians’ perspectives on communication about complementary and alternative medicine	40 Hispanic, 5 Non-Hispanic White, 48 Native American (72F, 21M) (n = 93)	SSI Thematic	√
Wullink, Veldhuijzen, van Schrojenstein, de Valk, Metsemakers & Dinant (2009) **[** 61]	Netherlands	Explore preferences of adults with intellectual disabilities based on positive and negative experiences of communication	12 adults with intellectual disabilities (8F, 4M) (ENR)	2 SSI & 1 FG Thematic	
Matthias, Bair, Nyland, Huffman, Stubbs, Damusb & Kroenke (2010) **[** 62]	USA	Compare patients’ experiences of communication with nursing staff and communication with physicians	18 adults with musculoskeletal pain and depression (11F, 7M) (ENR)	4 FGs Thematic	
Peek, Odoms-Young, Quinn, Gorawara-Bhat, Wilson & Chin (2010) **[** 63]	USA	Examine African American patients’ perceptions of the influence of race on physician-patient communication	51 African American with diabetes (42F, 9M)	24 SSI & 5 FGs Phenomenology	√
Yorkston, Johnson, Boesflug, Skala & Amtmann (2010) **[** 64]	USA	Explore patients’ experiences of communication about pain and fatigue	22 White, 1 Black adult with chronic pain (18F, 5M) (n = 23)	FG Thematic	
Jagosh, Boudreau, Steinert, MacDonald & Ingram (2011) **[** 65]	Canada	Understand patients attitudes, perceptions, and thoughts about their communication experiences	55 adults (10 French-speaking; 45 English-speaking; 3 Bilingual) (32F, 26M)	SSI Thematic	
Walseth, Abildsnes & Schei (2011) **[** 66]	Norway	Verify Haberma’s theory of communication according to patients’ perspective	12 adults (5F, 7M) (ENR)	SSI Thematic	
Black (2012) **[** 67]	USA	Explore elders' perspective of the influence of their beliefs on health care encounters	60 African American elders (30F, 30M)	SSI Thematic	√
Burton (2012) **[** 68 **]**	Guatemala	Explore the ways in which facework influences physician-patient interactions for Achi patients	24 Achi Aboriginal patients	SSI & observations Thematic	√
**Dahm (2012) [** 69 **]**	Australia	Explore the relationship between perceived time constraints, jargon use, and patient information-seeking	7 Non-Native English-speakers from Europe and Asia, 10 Native English-speakers (14F, 3M) (n = 17)	SSI Grounded Theory	√
**Hartley, Sutherland, Brown & Yelland (2012) [** 70 **]**	Australia	Explore women's views of care provided by physicians in the first 12 months postpartum	29 women (ENR)	SSI Thematic	
**Holmvall, Twohig, Francis & Kelloway (2012) [** 71 **]**	Canada	Explore patients' experiences of fairness and commitment in health care contexts	23 adults (15F, 8M) (ENR)	SSI Grounded Theory	
**Shannon, O'Dougherty & Mehta (2012) [** 72 **]**	USA	Explores refugees’ perspectives regarding communication barriers impeding on communication about war related trauma	37 Liberia, 3 Laos, 3 Asian, 4 Africa, 1 Bosnia, 3 South American (32F, 18M) (n = 50)	SSI Thematic	√
**Weber & Mathews (2012) [** 73 **]**	USA	Explore patients' perceptions of the quality of care delivered by a foreign international medical graduate physician	4 White, 5 Black, 1 Native American lower SES patients (6F, 4M) (n = 10)	SSI Thematic	√
**Bergman, Matthias, Coffing & Krebs (2013) [** 74 **]**	USA	Understand respective experiences, perceptions, and challenges both patients with chronic pain and physicians face communicating about pain	20 White, 4 Black, 2 Other chronic pain patients (2F, 24M) (n = 26)	SSI Thematic	
Claramita, Mubarika, Nugraheni, van Dalen & van der Vleuten (2013) **[** 75]	Indonesia	Examine cultural relevance of Western physician-patient communication style to Indonesian physician-patient interactions from the patients' and physicians’ perspective	20 Javanese patients (Indonesian)	SSI Grounded Theory	√
Cocksedge, George, Renwick & Chew-Graham (2013) **[** 76]	UK	Explore the use of touch in consultations from both physician and patient perspectives	10 White British, 1 other (n = 11)	SSI Thematic	
**Hughes (2013) [** 77 **]**	USA	Explore women’s experiences of communication about sexual health	13 White, 13 African American, 1 Native American women (n = 27)	SSI Thematic	
**Wilkinson, Dreyfus, Bowen & Bokhour (2013) [** 78 **]**	USA	Examine communication and interaction as experienced by patients and physicians	23 White, 3 Black intellectually disabled women (n = 27)	SSI Grounded Theory	
**Baumbusch, Phinney, & Baumbusch (2014) [** 79]	Canada	Explore the perspective of adults with intellectual disabilities on helpful interactions with their family physician	11 adults with intellectual disabilities (7F, 4M)	SSI Thematic	
Bayliss, Riste, Fisher, Wearden, Peters, Lovell, & Chew-Graham (2014) [80]	UK	Explore possible reasons why people from Black and ethnic minority groups may be less frequently diagnosed with chronic fatigue syndrome or myalgic encephalitis	6 Pakistani, 2 Indian, 2 Black British, 1 Other White patients with chronic fatigue syndrome (8F, 3M) (n = 11)	SSI Thematic	√
Marcinowicz Pawlikowska & Oleszczyk (2014) **[** 81]	Poland	Identify which aspects of GPs' behaviour are the most important for older people in their perception of the quality of the GP visits	30 patients over the age of 65 (18F, 12M)	SSI Thematic	
Matthias, Krebs, Bergman, Coffing, & Bair (2014) [82]	USA	Advance the understanding of communication about opioid treatment for chronic pain	7 African American, 23 White veteran patients with chronic pain (4F, 26M) (n = 30)	SSI Thematic	
Ritholz, Beverly, Brooks, Abrahamson, & Weinger (2014) [83]	USA	Explore perceptions of barriers and facilitators to diabetes self-care communication during medical appointments	34 patients with diabetes (82% non-Hispanic White) (41% female)	SSI Thematic	
**Rose & Harris (2014)** [84]	Australia	Explore the experiences of ethnically diverse patients with diabetes in receiving self-management support from GPs	11 Arabic-speaking migrants, 9 English-speaking migrants, 8 Vietnamese-speaking migrants (17F, 11M) (n = 28)	FG Phenomenology	√
Esquibel & Borkan (2014) [85]	USA	Explore ways in which opioid medication influences the doctor-patient relationship by exploring experiences of adults receiving opioid therapy and that of their physicians	21 patients receiving opioid therapy (13F, 8M)	SSI Thematic	
Melton, Graff, Holmes, Brown, & Bailey (2014) [86]	USA	Explore the experience of asthma patients in the management of their illness	4 African American patients with asthma (4F)	SSI Phenomenology	√
**Silver (2015)** [87]	Canada	Explore barriers and facilitators of patient-provider communication about patient searches for health information on the Internet	56 elderly patients (57% born in Canada) (30F, 26M)	SSI Grounded theory and thematic	

^a^UK: United Kingdom.

^b^UII: Unstructured individual interviews.

^c^√: Cultural aspects are discussed.

^d^ENR: Ethnicity not reported.

^e^SSI: Semi-structured individual interviews.

^f^USA: United States of America.

^g^FG: Focus group interviews.

^h^II: Individual interviews.

^i^SES: Socio-economic status.

### Patients’ Experiences of Communication: Three Major Concepts

**Fig 5:**
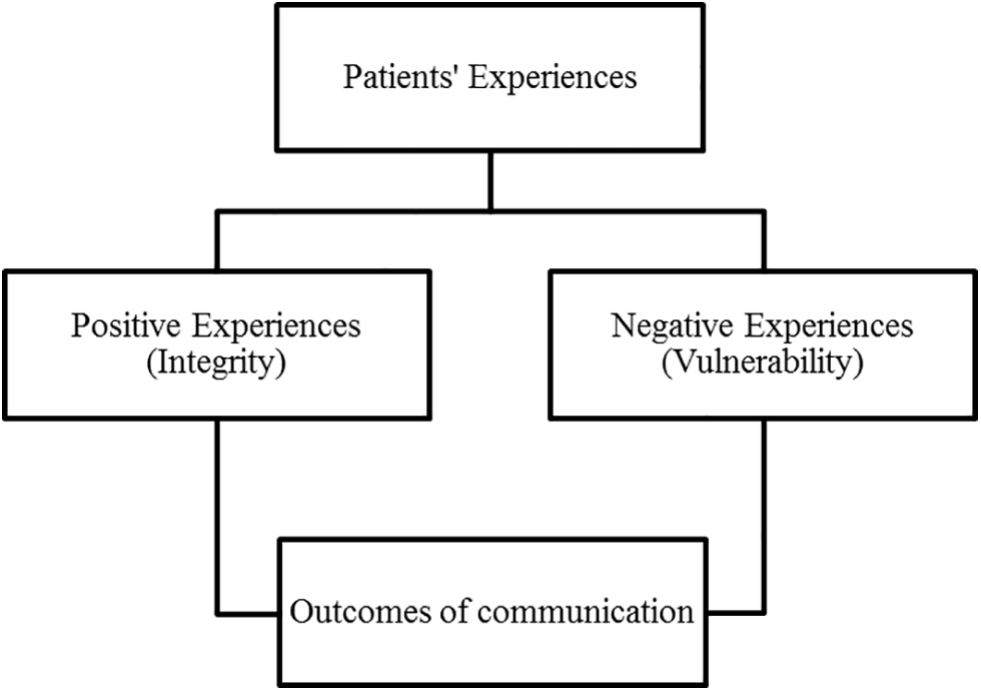
Summary of the three major concepts: positive experiences, negative experiences, and outcomes of such communication experiences.

Three major concepts emerged in response to the first research question: a) experiences evaluated negatively, b) experiences rated as positive, and c) outcomes of these communication experiences. Negative experiences raised by patients revolve around feelings of vulnerability related to enacting the patient role. Positive experiences generally relate to being treated with respect, thus allowing patients to maintain a sense of integrity. The third concept relates to repercussions of communication. Outcomes of communication vary depending on the quality of the communication experience. For the sake of succinctness, only a few examples of references supporting results are presented in this section. See Fig 5 for a summary of the major concepts.

### Negative Experiences: Vulnerability

Ninety-three percent of the studies (53 of the 57) reported results relating to the state of vulnerability associated to enacting the patient role. This concept seems to be omnipresent in the experience of communicating with PCPs. In fact, from 1995 to 2015, patients from a wide range of countries and micro-cultures report feeling vulnerable when communicating with PCPs.

This vulnerability stems from four overlapping and occasionally interdependent categories of experiences: disrespect, time constraints, dominance of biomedical culture, and helplessness (Table 2).

**Table 2:** Synthesis of the first concept: Negative experiences. Negative experiences discussed by participants are grouped in four categories: experiences of disrespect, time constraints, dominance of biomedical culture, and feelings of helplessness. Examples raised by participants for each category are presented.

Negative Experiences	Examples
Disrespect	Patients are ignored, interrupted, not taken seriously, not listened to, labelled and treated accordingly (discrimination). Patients’ expertise is dismissed, privacy not respected
Time Constraints	Patients feel unworthy of physician's time, like a burden, rushed (which limits what is brought up), dehumanized/depersonalized, stressed and limited in questions they can ask. Physicians’ behaviour is affected: asks less questions or more close-ended questions, uses more jargon
Time Constraints	Patients feel unworthy of physician's time, like a burden, rushed (which limits what is brought up), dehumanized/depersonalized, stressed and limited in questions they can ask. Physicians’ behaviour is affected: asks less questions or more close-ended questions, uses more jargon
Dominance Of Biomedical Culture	Patients feel that biomedical culture dominates: physicians decide what is discussed (mostly biomedical info), physicians do not get to know the patient in a holistic way, use incomprehensible medical jargon, patients’ attempts to discuss psychosocial information related to disease fail, lack of continuity of care
Dominance Of Biomedical Culture	Patients feel that biomedical culture dominates: physicians decide what is discussed (mostly biomedical info), physicians do not get to know the patient in a holistic way, use incomprehensible medical jargon, patients’ attempts to discuss psychosocial information related to disease fail, lack of continuity of care
Feelings Of Helplessness	Patients don't understand jargon, are too shy/intimidated to ask, need the physician to initiate conversation, fear disclosure of information (fear physician’s reaction), undergo intimate procedures without preparation (experience discomfort with physicians but must endure), experience anxiety and shame while intimate information disclosure/inquiry, experience shock when obtaining diagnosis depending on physician’s disclosure technique, need to expose oneself to many doctors, experience difficulty setting agenda and expressing symptoms
Feelings Of Helplessness	Patients don't understand jargon, are too shy/intimidated to ask, need the physician to initiate conversation, fear disclosure of information (fear physician’s reaction), undergo intimate procedures without preparation (experience discomfort with physicians but must endure), experience anxiety and shame while intimate information disclosure/inquiry, experience shock when obtaining diagnosis depending on physician’s disclosure technique, need to expose oneself to many doctors, experience difficulty setting agenda and expressing symptoms

#### Disrespect

This category relates to being treated with disrespect. This experience varies in intensity from patients reporting feeling as if they are not being taken seriously to patients recalling being stigmatized, mocked, and discriminated [46, 68]. Other examples raised were not listening to the patient or ignoring the patient, disrespecting the patient’s privacy (e.g. allowing interns to attend a consultation without warning the patient), interrupting the patient, dismissing the patient’s expertise regarding the illness, and not recognizing the patient as an autonomous and self-reliant individual [34, 41, 49, 62]. Consequently, such experiences led to patients not wanting to disclose or fearing disclosing important health information (e.g. psychological illness, sexual orientation, alternative medicine use) and patients developing other ways of expressing pain so it would be recognized and taken seriously, such as crying [34, 46, 88].

#### Time Constraints

Time constraints translate in the patient reporting feeling unworthy of the physician’s time, feeling like a burden, feeling rushed, not properly listened to, feeling stressed, not having the opportunity to tell their story, to ask questions, or to broach all issues [52, 58]. Time constraints limit discussion to physical symptoms and psychosocial aspects are neglected. Consequently, patients explain feeling dehumanized, and they compare their experience to being part of an assembly chain or feeling like a number [71, 74]. Physicians’ behaviours are also perceived to be more disagreeable in response to time constraints [82]. Physicians are perceived to ask fewer questions or ask more close-ended questions, seem disinterested, focus on the physical symptoms related to the disease, limit social talk, and tend to utilize jargon to rapidly explain the condition [45, 69, 89].

#### Dominance of Biomedical Culture

As described by patients, biomedical culture served to orient conversations towards physical symptoms and biological aspects of one’s condition and made it very difficult for patients to obtain or to discuss psychosocial information regarding their condition (e.g. emotions and impact of illness on their life) [47, 77]. Although some patients recall attempting addressing psychosocial issues and being dismissed, most patients report waiting for the physician to broach psychosocial aspects and report not feeling confident in raising such topics. Consequently, patients did not have the opportunity to tell the physician about pertinent psychosocial information relating to their illness [53, 90]. Without such information, it is difficult for the physician to have a global understanding of the patient’s psychosocial context and to tailor the treatment plan to the patient’s context.

In addition, the dominance of biomedical culture fosters an asymmetric physician-patient relationship which must be endured by patients. For instance, they describe physicians as leading the consultation, sometimes in a paternalistic way, by orienting the conversation towards physical symptoms and by leaving no room for psychosocial aspects related to the condition. Patients are left powerless and with little control, thus explaining their lack of confidence to broach psychosocial topics. Some patients even described the negative experience of feeling like a child because the physician behaved in such a paternalistic manner by deciding on the treatment plan, resorting to complicated jargon, and neglecting the patient’s expertise with regard to the illness [49, 55, 62, 89].

#### Helplessness

The dominance of biomedical culture could serve to partly explain the category of helplessness. Patients of all cultural and micro-cultural background reported feeling helpless during consultations. Events that triggered such feelings were: having to expose oneself and to disclose intimate information, undergoing intimate procedures, not being in control of the consultation while being stuck in an asymmetric conversation pattern, and having to repeat this experience often with a series of doctors. In addition, patients report not understanding the physician’s explanations and jargon and feeling too shy or intimidated to ask for clarifications, the obligation to disclose sensitive information (e.g. non-adherence) and the experience of fear and anxiety with regard to communicating about certain unavoidable health-related topics (e.g. sexuality, abuse), undergoing intimate procedures without being prepared by the physician, experiencing discomfort with certain doctors but having to undergo the examination, and difficulty expressing one’s symptoms and setting the agenda [33, 47, 50, 54].

### Positive Experiences: Preserving Integrity

Positive experiences were raised by 43 of the 57 studies (75%) and are attributed to certain relational skills, technical skills, as well as certain approaches to care privileged by the physician (Table 3).

**Table 3:** Synthesis of the second concept: Positive experiences. Positive experiences discussed by participants are grouped in three categories relating to physicians’ relational skills, technical competences, and approach to care. Examples raised by participants for each category are presented.

Positive Experiences	Examples
Physicians’ Relational Skills	Patients appreciate being treated with respect and dignity, an empathic, supportive, genuinely interested, friendly, compassionate, respectful, open-minded, accepting, non-judgemental, honest, frank physician, continuity of care (getting to know a physician), use of humour and use of appropriate touch by physician, (some patients appreciate) similarity with physician (e.g. gender, religious beliefs, and ethnicity).
Physicians’ Technical Competence	Patients appreciate a highly competent physician who takes time to investigate symptoms, ask questions, initiate sensitive topics, allows patients to ask questions, is attentive to detail, makes correct diagnosis, gives time to patient to talk, takes time to listen, and does not rush, carefully educates the patient, gives precise explanations, tailors word choice to patient, avoids jargon, uses images and text, gives enough information, takes action, orders appropriate tests, makes referrals when needed, extends consultation time, explains before doing (sensitive) procedures
Physicians’ Technical Competence	Patients appreciate a highly competent physician who takes time to investigate symptoms, ask questions, initiate sensitive topics, allows patients to ask questions, is attentive to detail, makes correct diagnosis, gives time to patient to talk, takes time to listen, and does not rush, carefully educates the patient, gives precise explanations, tailors word choice to patient, avoids jargon, uses images and text, gives enough information, takes action, orders appropriate tests, makes referrals when needed, extends consultation time, explains before doing (sensitive) procedures
Physicians’ Approach to Care	Patients appreciate when physicians tailor their approach to patients, perceive the patient in his or her context and gets to know the patient as an individual, consider patients’ health and illness representations, value the patient’s opinion and expertise towards the illness, focus on topics other than biomedical information. Majority of patients appreciate equality and partnership between patient and physician. Minority of patients appreciate hierarchical/paternalistic approach.
Physicians’ Approach to Care	Patients appreciate when physicians tailor their approach to patients, perceive the patient in his or her context and gets to know the patient as an individual, consider patients’ health and illness representations, value the patient’s opinion and expertise towards the illness, focus on topics other than biomedical information. Majority of patients appreciate equality and partnership between patient and physician. Minority of patients appreciate hierarchical/paternalistic approach.

#### Relational Skills

Patients raised many examples of positive communication experiences that were due to physicians’ satisfactory relational skills. Patients appreciated when their physicians were empathic, careful listeners, open-minded, non-judgemental, respectful, friendly, compassionate, and seemed genuinely interested [32, 65, 91]. These qualities made patients feel less ashamed to discuss sensitive topics and attenuated their fear of being judged [83, 87]. Some patients enjoyed when the physician used humour to alleviate the tension, others recall appreciating the use of appropriate touch (e.g. hand on shoulder), and some raised the general idea that perceived similarity to the physician made communication more agreeable [32, 43, 56, 59, 63, 76]. For instance, some men and women voiced preferring same gender physicians, some African Americans preferred African American physicians, and some patients with religious beliefs preferred communicating with a physician who shared concordant beliefs. Finally, the opportunity to upkeep the relationship and build on it over time (i.e. continuity of care) was often raised as positive [36, 38].

#### Technical Skills

Patients appreciate a competent physician who takes time to investigate, gives the patient time to talk and to ask questions, is attentive to details, asks further questions, initiates conversations about sensitive topics, provides enough information, and successfully educates the patient [45, 46, 48, 81]. Furthermore, patients report being grateful when physicians tailored their word choice, thus avoiding incomprehensible jargon, or took time to explain the condition by utilizing images and text, and ensured that the patient had understood [44, 55, 79, 90]. Finally, patients value physicians who take appropriate action, such as ordering appropriate tests, referring patients to proper specialists, and establishing a correct diagnosis [40, 56]. Such satisfactory technical skills may diminish feelings of vulnerability a patient may be experiencing. For instance, some men report that they felt more at ease and less intrusion when physicians took time to explain intimate procedures [42].

#### Tailored Approach to Care

Tailoring the approach to the patient and individualizing care seems to ensure a positive communication experience. A majority of patients emphasized the importance of feeling personally understood in one’s context and cared for in a biopsychosocial approach in contrast with being treated as a number [66, 70, 72, 86]. Furthermore, they appreciated when physicians valued their opinion and when they had an egalitarian relationship and a partnership with their physician [35, 71, 73]. Nevertheless, a minority of patients reported appreciating when physicians engaged in more paternalistic behaviours and enacted the role of the expert [40, 52]. In short, patients’ discourse suggests that the key to providing a positive communication experience to all patients is to tailor the approach to their preferences.

### Outcomes of Communication

**Fig 6:**
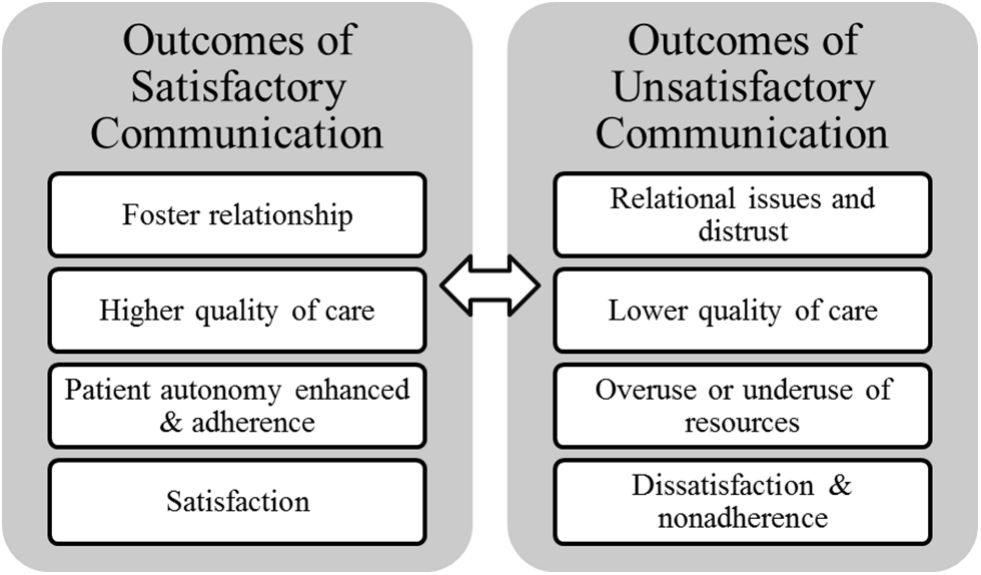
Outcomes of communication according to degree of satisfaction with communication experience.

The third concept relates to outcomes of communication as perceived by patients. Inverse outcomes were recalled by patients reporting mostly unsatisfying communication experiences compared to their satisfied counterparts (Fig 6).

#### Outcomes of Satisfactory Communication

Patients who experienced positive communication reported beneficial outcomes [65, 90, 92, 93] which are classified in four categories: a) fostering the relationship, b) higher quality of care, c) enhanced patient autonomy and adherence, and d) satisfaction (Table 4).

**Table 4:** Translation of the third theme: Outcomes of a Positive Communication Experience. Positive outcomes of communication experiences raised by participants are grouped in four categories: fostering relationship, higher quality of care, patient autonomy enhanced, and satisfaction and adherence. Examples raised by participants for each category are presented.

Positive Outcomes of Communication	Examples
Fostering the Relationship	Fosters trust and a satisfying relationship, helps create a sense of partnership
Higher Quality of Care	Proper referrals, patient satisfaction, disclosure of important health related information
Enhanced Patient Autonomy & Adherence	Feel more in control and responsible, more motivated to change and adhere to treatment, and to engage in more information-seeking behaviours
Satisfaction	Reduces feelings of vulnerability, relieves pain, stress, patients feel consoled, supported, cared for, valued, and welcomed

Satisfactory communication led to the development of a satisfactory relationship since positive experiences fostered trust and a sense of partnership [48, 68, 81]. Patients also evaluated care as being of higher quality since patients report being able to disclose important but sensitive information and thus equipping physicians to make proper referrals to address these issues [46, 55, 57]. Furthermore, patients report feeling more autonomous which leaves them with a feeling of control and responsibility, being more motivated to change and to adhere to the treatment plan and more empowered to ask questions and seek more information [60, 66, 94]. Finally, patients report being satisfied as illustrated by reduced feelings of vulnerability, a relief from stress and pain, as well as patients feeling consoled, supported, cared for, valued, and welcomed [40, 61, 65, 70].

#### Outcomes of Unsatisfactory Communication

Patients also discussed harmful consequences ensuing from negative communication experiences [68, 75, 77, 78, 95] which are grouped in four categories: a) relational issues and distrust, b) lower quality of care, c) overuse or underuse of resources, and d) dissatisfaction and non-adherence (Table 5).

**Table 5:** Translation of the third theme: Outcomes of a Negative Communication Experience. Negative outcomes of communication experiences raised by participants are grouped in four categories: relational issues and distrust, lower quality of care, overuse or underuse of resources, and dissatisfaction and non-adherence. Examples raised by participants for each category are presented.

Negative Outcomes of Communication	Examples
Relational Issues & Distrust	Patients experience difficulty establishing a high quality relationship, distrust in physician, lack of faith in the physician’s capacity to help
Lower Quality of Care	Patients do not receive enough information to understand illness in all its facets, disclose important health-related information, not all issues are addressed, receive proper referrals, receive a treatment plan suited to their reality, receive enough information about treatment plan, or the treatment plan is badly explained
Overuse or Underuse of Resources	Patients seek a second opinion or consult elsewhere for the same issue, avoid consulting for future issues or consult only if perceived as an emergency, delay follow-ups, change physicians
Dissatisfaction & Non-Adherence	Patient are unsatisfied, frustrated, feel unrecognized, and disapprove of physician's approach, less motivated to comply or adhere to treatment

Relationships were hard to establish with unsatisfactory communication as patients reported distrust and lack of faith in the physician [35, 39]. Furthermore, quality of care was reported as inferior since patients had not had the chance to disclose all pertinent health-related information, therefore proper referrals were not given and the treatment plan did not suit the patient or was poorly explained [67, 70, 77]. Following an unsatisfactory consultation, patients tended to overuse resources as patients sought a second and sometimes a third opinion, consulted elsewhere in order to attempt understanding their condition, searched for a new family physician, thus congesting health care systems for the same issue [40, 64, 75, 82]. On the contrary, some patients also reported avoiding consulting in the future and would delay follow-ups to avoid such negative experiences [58, 68, 80, 87]. Finally, patients expressed dissatisfaction and reported feeling frustrated, unheard, unrecognized, in disapproval with the physician’s approach and more severely, unmotivated to comply with the treatment plan [64, 65, 75].

### Factors Influencing Ethnic Minority Patients’ Experiences

In order to address the second research question, studies reporting on EMPs experiences were compared to studies investigating the general patient population. Although experiences were similar among both groups of patients, EMPs did raise distinctive experiences which relate to four categories of factors: a) language barriers, b) discrimination, c) differences in values and beliefs, and d) acculturation issues. These factors exerted mostly a negative influence on communication (see Tables 6 and 7 for a summary).

**Table 6:** Overview of studies discussing specific factors affecting EMPs’ experiences (n = 23): author, year, country of publication, sample, aim, and synthesis of specific factors.

Author (Year)	Country	Sample	Aim	Synthesis of Specific Factors Affecting EMPs
Bowes & Domokos (1995) **[** 32 **]**	UK^a^	20 Pakistani born women	Explore South Asian women's experiences and use of health services	Language barriers are problematic, challenging to find a professional interpreter, feel labelled and treated according to stereotypes (e.g. relating to traditional clothing)
Thom, Campbell & Alto (1997) **[** 35 **]**	USA^b^	29 patients (1 FG^c^ Hispanics, 1 FG African Americans, 2 FG White Americans)	Identify physician behaviours that foster trust	EMPs^d^ report more disrespect and discrimination and more negative experiences (e.g. death due to medical mistakes) than Whites. Whether real or perceived discrimination, it affects their trust in physician.
Rodriguez, Bauer, Flores-Ortiz, Szkupinzki-Quiroga (1998) **[** 36 **]**	USA	14 Hispanic and 14 Asian women (n = 28)	Identify provider related factors that may affect physician-patient communication about abuse for immigrant women	Abused immigrant women's discourse is similar to White patients’ discourse with regard to disclosing sensitive info (e.g. need to be asked, need to be empathic).
**Ali, Atkin & Neal (2006) [** 25 **]**	UK	7 White British and 16 South Asian	Understand the ways in which White and South Asian patients communicate with white physicians	South Asian patients experience language barrier. South Asians prefer White doctor because less importance is attributed to social hierarchy and authority in the UK. Whites and South Asians have different evaluations of similar experiences (e.g. South Asians don't perceive social talk as pertinent to a consultation). South Asians more critical of care than Whites.
**Cant & Taket (2006) [** 46 **]**	UK	18 White, 5 Black and other ethnic minority (n = 23)	Explore lesbians’ and gays’ experiences of primary care	Gay or lesbian EMPs feel their ethnic minority identity intersects with their homosexual identity. For some, it is felt as potentially increasing their risk and experience of discrimination.
**Towle, Godolphin & Alexander (2006) [** 48 **]**	Canada	26 Aboriginals	Understand the complexity of physician-patient communication in Aboriginal communities	Aboriginals experience strong discrimination and distrust in physicians. Feelings linked to historical context and previous historical trauma. Time constraints seem to afflict Aboriginals even more since concept of time is different for Aboriginals; giving someone time is respectful as it shows the other person that he or she is worthy.
Abdulhadi, Shafaee, Freudenthal, Östenson & Wahlström (2007) **[** 50 **]**	Oman	27 Omani	Explore views of type 2 diabetic patients regarding medical encounter	Social hierarchy and respect for authority seems highly valued and prescribed in Oman and patients do not appreciate that; some even prefer a physician from another culture in order to reduce feelings of inferiority.
Fagerli, Lien & Wandel (2007) **[** 52 **]**	Norway	16 Pakistani born	Explore patients’ experiences of medical encounters	Pakistani EMPs in Norway experience language barrier. Seems like acculturation experiences influence communication experiences; more acculturated patients (e.g. workers, fluent in Norwegian) rate experiences as more positive than less acculturated patients.
Kokanovic & Manderson (2007) **[** 53 **]**	Australia	8 Chinese, 8 Indian, 8 Pacific Island, and 8 Greeks	Describe the way patients in an Australian setting are told of diabetes	EMPs' discourse resembles that of general patient population (e.g. short consultations, do not understand complicated jargon used to explain condition, appreciate when offered psychosocial information on diabetes). Experiences seem homogenous between immigrant groups but small sample, thus difficult to evaluate.
Lowe, Griffiths & Sidhu (2007) **[** 54 **]**	UK	17 Pakistan-born, 2 UK born but Pakistani origin	Explore attitudes and experiences of South Asian women towards contraceptive service provision	Pakistani women experience language barrier, difficulty accessing professional interpreters, obliged to turn to family and friends. They feel uncomfortable discussing certain topics in front of family and friends; therefore, they do not receive the information they need. Preference for female doctors. Problems due to cultural values such as respect for authority and negative consequences of ethnic match (e.g. for religious reasons, some physicians refuse to discuss contraception and women feel powerless in broaching the issue).
Julliard, Vivard, Delgado, Cruz, Kabak & Sabers (2008) **[** 57 **]**	USA	28 Hispanic women (8 born in US, 20 born in South or Central America)	Clarify which conditions reinforce nondisclosure of health information in clinical encounters between Latina patients and their physicians	Language barriers are problematic; difficult to disclose sensitive information when working with untrained interpreters because of privacy issues, women are frustrated and embarrassed. Values and belief differences; sexual issues are a big taboo. For Hispanics, need to maintain harmonious relations, so patients are fearful to disclose sexually transmitted diseases or abuse because of risk of destroying relations. Acculturation influence; women born outside the US prefer female physicians and need established relationship, trust, and warmth, whereas US-born patients understand that physician’s role is mainly to heal, therefore, not as much importance attributed to a warm relationship.
Nguyen, Barg, Armstrong, Holmes & Hornik (2008) **[** 58 **]**	USA	20 Vietnamese immigrants	Examine elements of physician-patient cancer communication from the viewpoint of older Vietnamese immigrants	Cultural belief; if you talk about an illness, it will develop. Physicians do not take belief into consideration when discussing prevention of illness. Language barrier is problematic and patients are not aware that the system needs to provide an interpreter. Patients accept the paternalistic model. Although they feel they do not have enough information to understand tests and procedures, they do not adopt the active patient role since for them it is the physician's role to initiate conversations.
Shelley, Sussman, Williams, Segal & Crabtree (2009) **[** 60 **]**	USA	40 Hispanics, 5 Non-Hispanic White, 48 Native Americans	Compare patients’ and physicians’ perspectives on communication about complementary and alternative medicine	EMPs’ and Whites’ discourse is similar except that for EMPs, alternative medicine seems to be more related to their cultural traditions, therefore they do not think the physician would understand or that it concerns the physician.
Peek, Odoms-Young, Quinn, Gorawara-Bhat, Wilson & Chin (2010) **[** 63 **]**	USA	51 African Americans	Examine African American patients’ perceptions of the influence of race on physician-patient communication	African American patients experience discrimination based on physicians' stereotypes (e.g. do not have as much time to talk as White patients, feel as if the physician did not explain because believed that Blacks would not understand). Most patients agree that it is best to consult a Black physician, ethnic match is positive.
Black (2012) **[** 67 **]**	USA	60 African American Elders	Explore elders' perspective of the influence of their beliefs on health care encounters	Black elderly patients say they feel more discriminated on the basis of their old age than on their skin colour, although many had examples of discrimination linked to their skin colour. Patients do not like that physicians do not inquire or know about their health and illness representations and cultural beliefs
Burton (2012) **[** 68 **]**	Guatemala	24 Achi (Aboriginal) patients	Explore the ways in which facework influences physician-patient interactions for Achi patients	Aboriginals experience serious discrimination (e.g. being ignored, physically and psychologically abused, and neglected). Physicians do not take into account cultural beliefs and norms when communicating (e.g. speak directly to patient and criticize their habits, for Achi, need to communicate indirectly to remain polite and respectful).
Dahm (2012) **[** 69 **]**	Australia	7 Non-Native English speakers from Europe and Asia, 10 Native English speakers (n = 17)	Explore relationship between perceived time constraints, jargon use, and patient information-seeking	EMPs feel the same about jargon and time constraints as general patient populations, but they do not focus and complain as much about short consultation times because consultations are even shorter in their countries of origin.
Shannon, O'Dougherty & Mehta (2012) **[** 72 **]**	USA	37 Liberia, 3 Laos, 3 Asian, 4 Africa, 1 Bosnia, 3 South American (n = 50)	Explores refugees’ perspectives regarding communication barriers impeding on communication about war related trauma	Differences in health representations; EMPs did not perceive war-related symptoms or emotional distress as health-related. Discourse is similar to White patients regarding disclosure of sensitive information; they believe they should defer authority to physician and should not be the one to initiate such conversations.
Weber & Mathews (2012) **[** 73 **]**	USA	4 White, 5 Black, 1 Aboriginal	Explore patients' perceptions of quality of care delivered by a foreign international medical graduate physician	Majority of patients evaluate experience as positive because of status equalization effect between ethnic minority physician and patients. However they mention language barriers associated to foreign physician's accent.
Claramita, Mubarika, Nugraheni, van Dalen & van der Vleuten (2013) **[** 75 **]**	Indonesia	20 Javanese patients (Indonesian)	Examine cultural relevance of Western physician-patient communication style to Indonesian physician-patient interactions from the patients' and doctors' perspective	Majority of patients in non-western country are not satisfied with paternalistic styles but are less able to defy this style because of predominance of collectivist values to maintain harmonious relationships and respect for authority.
Bayliss, Riste, Fisher, Wearden, Peters, Lovell, &Chew-Graham (2014) [80]	UK	6 Pakistani, 2 Indian, 2 Black British, 1 Other White	Explore possible reasons why people from Black and ethnic minority groups may be less frequently diagnosed with chronic fatigue syndrome or myalgic encephalitis	Language barrier is a problem for patients in expressing their symptoms and in understanding the physician. Some turn to professional interpreters, however, the interpreter does not always understand the patient’s dialect. Others bring family members or notes written by a community member. Patients feel physicians have negative stereotypes of their culture (e.g. lazy and complainer) and treat the patients accordingly.
Rose & Harris (2014) [84]	Australia	11 Arabic-speaking migrants, 9 English-speaking migrants, 8 Vietnamese-speaking migrants	Explore the experiences of ethnically diverse patients with diabetes in receiving self-management support from GPs	Patients feel they are not provided with enough culturally tailored advice. Some patients also aim to protect the relationship with their physician, although they dislike the paternalistic style.
Melton, Graff, Holmes, Brown, & Bailey (2014) [86]	USA	4 African American	Explore the experience of asthma patients in the management of their illness	Patients feel discriminated against based on their skin colour and historical tensions between African Americans and White Americans influence the way patients experience the consultation with physicians.

^a^UK: United-Kingdom.

^b^USA: United-States of America.

^c^FG: Focus group interviews.

^d^EMPs: Ethnic minority patients.

**Table 7:** Specific factors influencing EMPs’ experiences in communicating with a physician (language barriers, discrimination, differences in values and beliefs, and acculturation issues).

Specific Factors Affecting EMPs’ Experiences	Examples
Language Barriers	Difficulty accessing professional interpreters, obliged to turn to friends and family members, discomfort when consulting with untrained interpreters, some topics are not addressed with untrained interpreters
Discrimination	Based on their cultural belonging, treated according to physicians’ stereotypes
Differences in Values and Beliefs	Differences in health and illness representations (e.g. emotional distress is not health-related), differences in beliefs regarding respect for authority and need to maintain harmonious relationships; patients remain silent and do not contradict physicians, differences in time concepts
Acculturation Issues	More acculturated to host culture experience less language barriers and understand patient role in the host culture

#### Language Barriers

Difficulties associated to language barriers were discussed by EMPs and impeded on communication [25, 52, 80]. EMPs describe the challenge associated to accessing a professional interpreter [32, 58]. Consequently, some patients turned to informal interpreters (e.g. child, husband, or friends). Although a minority of EMPs raised feeling comfortable with informal interpreters [54], most patients reported feeling embarrassed, guilty, and uncomfortable when consulting with informal interpreters [32, 54, 57]. Moreover, patients feel the presence of these interpreters constrains discussion about sensitive topics, mental health topics are avoided, and disclosure of intimate information is difficult or impossible. In short, consulting without professional interpreters or with informal ones made communication challenging and sometimes impossible. As a result, patients were at risk of receiving inadequate care.

#### Discrimination

EMPs raised experiences of discrimination based on their cultural belonging [80]. For instance, African Americans felt they were treated according to the stereotype of being unintelligent, being work shy and lazy, and having an unhealthy diet, and being poor [63, 86]. For Pakistani-born migrants in Europe, some felt their traditional clothing impacted on the consultation with physicians acting surprised when these women proved to be intelligent and able to follow the consultation [32]. Some comparative studies allow stipulating that discrimination is a particularity of communication between EMPs and physicians. For instance, a focus group study conducted in the United-States illustrates that African Americans and Hispanics raise experiences of discrimination that remain absent from White Americans’ discourse [35].

Studies targeting Aboriginal populations conclude that discrimination is experienced in fair amounts by these EMPs. Marked distrust, described as ensuing from historical trauma, is obvious in their discourse and ongoing experiences of discrimination makes access to care difficult and impedes on help-seeking behaviours [48, 68]. For instance, Aboriginals in Guatemala report experiencing serious discrimination such as being mocked, abused, and insulted because of their indigenous background [68]. Aboriginals in Canada report feeling discriminated against since their selection of clinics to attend is restricted and they feel as if physicians treat them as inferior and talk down to them [48].

#### Differing Values and Beliefs

Studies raised specific examples of communication difficulties due to differences in beliefs and values. For instance, EMPs’ health and illness representations sometimes differed from the physicians’ and led to communication difficulties. Some Vietnamese patients believed that speaking about an illness would engender its development [58]. This belief was not considered or understood by physicians. Consequently, physicians attempted to discuss prevention measures for certain illnesses which left Vietnamese patients in states of fear or distress. Moreover, these patients may remain silent about recently appeared symptoms for fear of causing the illness to progress faster, thus impeding on early detection of illness. Another American study showed that for some African, South American, and Asian refugees in the United-States, emotional distress is not perceived as health-related, therefore, psychological distress is not discussed with physicians [72].

Differences in values and beliefs relating to respect for authority, the need to maintain harmonious relationships, time conceptions, and communication norms also seemed to engender communication difficulties. For some EMPs, the need to show respect for authority is much higher and the patient must avoid conflict at all cost with the physician [54, 75]. Therefore, patient participation is minimal. Despite the wish of some EMPs to be recognized as experts of their illnesses, most reported remaining passive in order to respect authority and to preserve the relationship with the physician [84]. Regarding the need to maintain harmonious relationships, Hispanics in the United-States explain that certain topics, such as sexuality, are taboo because they could put relationships at risk (e.g. disclosing abuse) [57]. Concerning beliefs about time, Canadian Aboriginals believe it is a sign of respect to give someone time and to get to know the person [48]. This conception clashes with mainstream Canadian culture and Aboriginal patients report harmful consequences of time constraints, such as distrust. Concerning communication norms, for Aboriginals in Guatemala, direct communication is interpreted as rude, insulting, and careless [68]. These patients thus report negative experiences when communicating with physicians in Guatemala who disregard Aboriginal communication norms. Finally, for some South Asian patients in the United-Kingdom, social talk initiated by the physician is perceived as intrusive and is therefore unappreciated [25].

#### Acculturation Issues

Although previous factors exerted mainly a negative influence on communication, acculturation may exert both positive and negative influences. Acculturation is a process which results from the contact between two cultures [96]. Following this contact, changes may be brought upon one’s cultural identity. For instance, some EMPs may identify to both their culture of origin and to the host culture while others remain affiliated only to their origins.

Some studies suggest that individual acculturation profiles may influence communication experience. For instance, a Norwegian study shows that EMPs rate their experiences differently according to their level of integration in the Norwegian society [52]. More precisely, the most integrated appreciated social talk, inquiry about psychosocial information, and being responsible for their care. In opposition, the less integrated did not appreciate when their opinion was solicited since they do not possess medical expertise, when physicians inquired about personal information since they perceive such information to be unrelated to medical issues, and disliked being given autonomy and responsibility. Another study conducted in the United-States shows that Hispanic migrants report the need to develop a warm relationship with their physicians before they feel safe sharing private information. On the other hand, United-States-born Hispanics seem to understand that the physician’s primary role in the United-States is to heal; therefore, they attach less importance to developing a warm relationship [57]. Finally, another study suggests that patients who are more acculturated to the host culture have more knowledge of the host culture’s language and escape dealing with language barriers [25].

In short, EMPs’ individual acculturation profile may exert an influence on communication experiences and must not be neglected. The positive influence of acculturation to the host culture is probably due to EMPs learning and adhering to the patient role as prescribed in the host country [52]. In contrary, the less acculturated EMPs remain unsure of their role as patients in host countries and thus evaluate the experience as unpleasant [52].

### Factors Influencing Ethnic Majority Patients’ Experiences

In order to address the third research question, studies exploring experiences of ethnic majority patients belonging to other minority or micro-cultural groups were compared to EMPs’ and to the general patient population’s experiences. Similarly to EMPs, patients belonging to other minority or micro-cultural groups also experienced additional distinctive communication difficulties (Table 8). These micro-cultural groups include, among others, patients with psychiatric disorders, religious patients, adults with intellectual disabilities, women going through menopause, and chronically ill adolescents.

**Table 8:** Specific factors related to micro-cultural belonging that influence ethnic majority patients’ experiences of communication.

Micro-cultural Aspects Influencing Ethnic Majority Patients’ Experiences	Examples
Discrimination	Patients feel they are labelled according to their differences and treated according to stereotypes
Differences in Values and Beliefs	Patients feel their religious and spiritual beliefs are dismissed as well as their beliefs concerning alternative medicine

These patients also experienced disrespect in higher proportion and intensity than the general patient population. For instance, adults with intellectual disabilities and adolescents with chronic illnesses report stronger feelings of being dismissed and unrecognized as autonomous individuals [38, 41, 94]. Examples of physicians’ disrespectful behaviours are addressing parents and support workers about the patient without the patient’s permission, making eye contact and talking to accompanying adults and not to the patient, and addressing the patient in childish ways. Furthermore, psychiatrically ill patients [39, 44, 46] and menopausal women [40] also experience being labelled and treated accordingly, homosexual patients report being the target of inappropriate and rude remarks regarding their sexual orientation [46], Elderly patients report experiencing frequent and unpleasant ageism [67], and patients receiving or in need of opioid therapy feel they are instantly labelled as drug addicts and feel they have to battle with the physician to defend their need for the treatment [82, 85].

Differences in values and beliefs and its negative effect on communication were also raised. For instance, some patients with different religious orientations experienced physicians dismissing their beliefs or avoiding such topics of conversation, thus acting as a barrier to building a satisfactory relationship [43]. Experiences of alternative medicine users are similar as they report feeling their beliefs are dismissed and thus fear discussing such topics [49].

## Discussion

The main objective of the current systematic review was to synthesize qualitative studies exploring patients’ experiences in communicating with PCPs. The secondary objective was to examine specific factors influencing EMPs’ experiences. The third objective was to explore specific factors raised by ethnic majority patients to gain a better understanding of micro-cultural influences on patients’ experiences. To our knowledge, this is the first systematic review of qualitative studies investigating patients’ experiences in communicating with a PCP.

Surprisingly, the first study to explore patients’ experiences in communicating with a primary care physician was published in 1995. As depicted in Fig 2, only 3 studies were published before the year 2000 and as of 2002, a steady interest in patients’ experience is apparent. This increase in interest would concord with, and could partly be due to, the popularisation of the patient-centered care approach which was developed during the 1980s but became central to researchers and health professionals only a few years later [97]. It is also possible that the increase in publications concords with the gradual acceptance and opening to qualitative research methods in medical research.

Regarding the first objective, patients reported both negative and positive experiences of communication which lead to either beneficial or harmful outcomes. Negative experiences relate to feeling vulnerable due to experiences of disrespect, time constraints, dominance of the biomedical culture, and helplessness. Although patients reported more negative experiences, they also recalled positive experiences relating to the opportunity to preserve one’s sense of integrity by being treated with respect by an empathic and competent physician who is capable of individualizing care. Outcomes of communication depended on the patient’s evaluation of the quality of the interaction. Patients raised positive outcomes, such as being satisfied and motivated to adhere to the treatment, and negative outcomes such as feeling frustrated and experiencing distrust in the physician.

### Counterbalancing Negative Experiences

**Fig 7:**
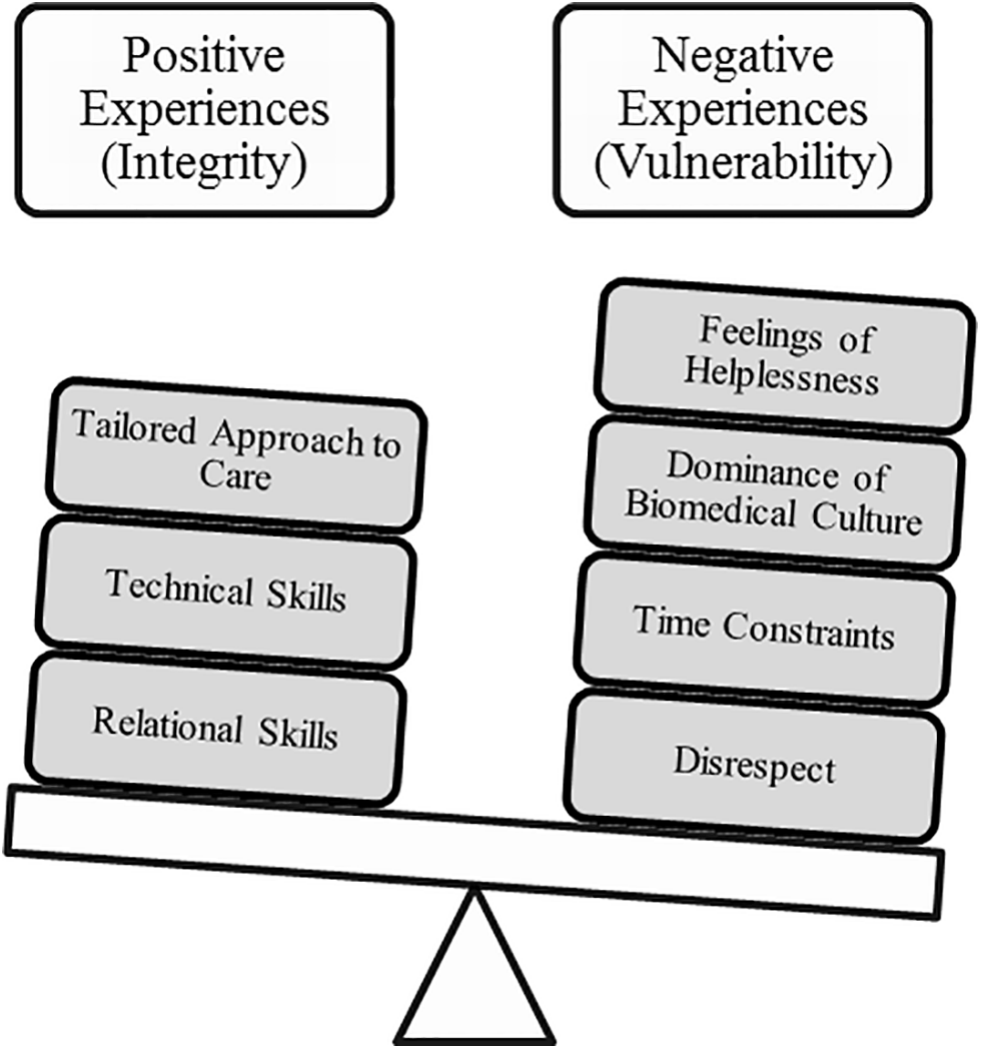
Relationship between positive and negative experiences: A balance metaphor.

The meta-ethnography method encourages the researcher to synthesize studies in a fashion that can provide a line of argument illustrating the relationship between concepts. In order to better understand the relationship between the three major concepts, a balance metaphor is depicted below (see Fig 7). Positive experiences are weighed on one side of the balance and negative experiences on the other. As suggested by findings, negative experiences weigh more (i.e. are more frequent) than positive experiences. Depending on the balance’s position following a consultation, the patient may report more positive or negative outcomes. This metaphor implies that positive communication experiences could offset or alleviate the unfavorable outcomes ensuing from negative experiences.

This relationship between vulnerability and integrity has been previously discussed [24]. Irurita [24] reports that hospitalized patients in Australia discussed feeling vulnerable due to losing control over their bodies but raised strategies they could engage in to preserve a sense of integrity. The author however concludes that strategies patients can engage in are limited and that to preserve patients’ integrity it is the responsibility of healthcare providers to engage in behaviours allowing the patient to remain in control and active in the care process.

Conversely, other authors underline the benefits of interventions aimed at patients to help them develop skills to better engage in communication in the health care process [85, 87]. These interventions prove to be helpful to patients and provide another avenue to ameliorating communication in health care. Interventions should indeed be aimed both at the patient and the physician in order to optimize care.

The context of physician-patient relationship can account for such ubiquitous feelings of vulnerability [98]. Power, expertise, and authority are attributed to the physician, while the patient is left without control and obliged to cooperate. Considering such an asymmetric relationship it is unsurprising that patients are left feeling vulnerable and that the physician has the duty and power to help patients retain a sense of integrity in the process. Indeed, results of this review show that despite some patients’ efforts, it seems to be difficult to preserve a sense of control partly due to the dominance of biomedical culture in medical consultations.

### Cultural Particularities

Findings relating to the second objective show that specific factors make the task of communicating with physicians more challenging for EMPs. It is the presence of these additional factors that could explain the higher rates of communication difficulties in intercultural dyads. These factors mainly exert a negative influence on communication and are classified in four categories; language barriers, discrimination, differences in values and beliefs, and acculturation issues. These categories resemble those raised by Schouten and Meeuwesen [6] in their review of communication difficulties in intercultural dyads. Interestingly, their review included only observational studies but results from the current review suggest that patients’ actual experiences concord with the accounts of observational studies.

Findings relating to the third objective show that some ethnic majority patients also experienced additional distinctive communication difficulties. Neuliep’s [16] contextual approach of intercultural communication can shed light on these findings. According to this approach, all interactions are nested within interconnected contexts which shape the communication. The first context is the general cultural context; all interactions take place within a general culture (e.g. national culture) which shares beliefs, norms, and rules. Within this context, Neuliep [16] introduces the micro-cultural context which also influences communication. Micro-cultures refer to all groups within the general cultural group who possess different beliefs, traditions, and behaviours than the general culture. One can belong to many micro-cultures depending on, for instance, one’s sexual identity, profession, gender, and age.

It is precisely these micro-cultures which serve to nuance some ethnic majority patients’ experiences, since their belonging to other micro-cultural groups influences their experiences of communication. This finding confirms the stipulation that all medical encounters are in fact intercultural. Communication in medical settings should be understood within its context, and cultural and micro-cultural influences should be taken into account. These micro-cultural groups seem to also be afflicted with stronger vulnerability from the outset. This affliction on EMPs and other micro-cultural groups can be depicted by the balance metaphor; an additional weight presses down on the negative experience side. For these patients, it is possible that it would take more consideration and sensitivity from the physician to make positive experiences offset harmful consequences of negative experiences.

### Strengths, Limitations, & Future Research

This review confirms the pertinence of qualitative methods for obtaining rich and detailed accounts of patients’ experiences in communication. The consistency in methods used by studies allows for rigorous comparison of results (46 used individual interviews and 14 used focus group interviews). Moreover, by including studies with heterogeneous patient samples, this review achieved one aim of qualitative research; it managed to identify trends across a diversity of participants in order to gain a general understanding of the experience [99].

In spite of these strengths, the current review and included studies present some limitations. First, only French and English articles were eligible. Second, studies published in books or other means than journals have not been examined since only peer-reviewed publications were targeted. Second, although diversity in samples allowed extracting general trends in experiences, generalizations to all types of patients must be made cautiously. Third, the preponderance of negative experiences over positive experiences may be due to a research bias towards investigating difficulties. Future studies should explore positive experiences in order to provide concrete examples of appreciated communication experiences.

Furthermore, some limitations concerning culture restrict our understanding of cultural aspects. For instance, a majority of studies (59.6%) neglected considering culture’s influence on patients’ experiences, despite previous evidence demonstrating culture’s potential influence on communication [6, 7, 100]. Moreover, a majority of the studies exploring cultural aspects omitted to present a definition of the terms and concepts used, which in turn limits the interpretation of their results. Additionally, designs used by studies that do examine cultural aspects of patients’ experiences limit our capacity to differentiate context from cultures or micro-cultures. Therefore, knowledge relating to intercultural communication remains nebulous. Future studies should undertake in-depth investigations of EMPs’ experiences by privileging intercultural comparative methods as they would allow differentiating context from culture. For instance, studies could compare migrants’ experiences of communication with a physician with those of locals. Future research should also consider the influence of EMPs’ individual acculturation profile on communication experiences, since current findings suggest this profile can serve to nuance such experiences.

Considering that micro-cultures can also influence communication, all medical encounters could be characterized as intercultural. Future research should investigate if existing intercultural communication approaches are applicable to all medical encounters. Better still, it would be pertinent to compare EMPs’ experiences with those of patients who are also perceived as different on the basis of their belonging to certain micro-cultures. This would allow the development of an encompassing theory and approach based on relations to all types of differences and not only ethnic differences.

## Conclusion

In conclusion, universal aspects of incarnating the patient role have been uncovered. Vulnerability will likely persist to be part of patients’ experiences due to the asymmetric physician-patient relationship. However, physicians should strive to treat patients with dignity and respect in order to alleviate the harmful effects of negative experiences. Physicians should also be more considerate and sensitive when communicating with EMPs and other micro-cultural groups as they seem to be experiencing more vulnerability from the outset.

### Practice Implications

Previous authors [24, 26] underlined the necessity of considering the patient’s experience in order to develop interventions aimed at adapting communication in healthcare. By considering patients’ experiences across 57 studies, this review provides clear and concrete results which can help tailor interventions to patients and will ensure more satisfactory experiences. Findings provide concrete examples of both positive and negative experiences which can inform healthcare providers regarding appreciated communication behaviours and disagreeable behaviours which may need to be modified. The balance metaphor and the concepts of integrity and vulnerability provide medical students and staff with concepts that are easily understandable and relatable and may facilitate the exercise of putting oneself in the patients’ shoes. Such concepts and metaphors may be used to render communication skills teaching more accessible and relatable. Finally, findings underline the necessity for physicians to remain aware of and sensitive to specific factors (cultural and micro-cultural) at play during communication. It is thus imperative that medical students be exposed throughout their training to these factors and their influence in order to overcome the neutralizing effect of medical socialisation. Healthcare workers, on the other hand, should reflect on these factors and keep them in mind when intervening with all types of patients.

## Supporting Information

S1 PRISMA Checklist PRISMA Checklist for systematic reviews. (DOC)

S1 Table Quality evaluation of included studies according to three domains of the COREQ checklist: research team and reflexivity, study design, and analysis and findings. Sub-categories of each three domain were selected as evaluation criteria. Studies presented good quality when a checkmark is indicated in at least one category of each domain. All but 3 studies had a checkmark in all three categories. These three studies failed to elaborate on the domain of Research Team and Reflexivity.
(DOCX)
