# Supplementary material for: A Systematic Review of Patients’ Experiences in Communicating with Primary Care Physicians: Intercultural Encounters and a Balance between Vulnerability and Integrity
Source: PLoS One. 2015 Oct 6;10(10):e0139577. doi: 10.1371/journal.pone.0139577 (PMC4594916; doi:10.1371/journal.pone.0139577)
Supplement: S1 Table — Sub-categories of each three domain were selected as evaluation criteria. Studies presented good quality when a checkmark is indicated in at least one category of each domain. All but 3 studies had a checkmark in all three categories. These three studies failed to elaborate on the domain of Research Team and Reflexivity. (DOCX) [file pone.0139577.s002.docx]

| Author (Year) | Research Team and Reflexivity | | Study Design | | | | | Analysis and Findings | | |
| --- | --- | --- | --- | --- | --- | --- | --- | --- | --- | --- |
|  | Personal Characteristics | Relationship with participants | Method | Sampling | Size | Setting : Data collection | Description of sample | Coding procedure | Quotations presented | Clarity of major concepts |
| Bowes & Domokos (1995) | x | x | x | x | x | x | x | x | x | x |
| Punamaki & Kokko (1995) | x | x | x | x | x | x | x | x | x | x |
| Johansson, Hamberg, Lindgren & Westman (1996) | x |  | x | x | x | x | x | x | x | x |
| Thom, Campbell & Alto (1997) | x |  | x | x | x | x | x | x | x | x |
| Rodriguez, Bauer, Flores-Ortiz, Szkupinzki-Quiroga (1998) | x |  |  | x | x |  | x | x | x | x |
| Pollock & Grime (2002) |  |  | x | x | x | x | x | x | x | x |
| Beresford & Sloper (2003) |  | x | x | x | x | x | x | x | x | x |
| Gask, Rogers, Oliver, May & Roland (2003) |  |  | x | x | x | x | x | x | x | x |
| Walter, Emery, Rogers & Britten (2004) | x |  | x | x | x | x | x | x | x | x |
| Ziviani, Lennox, Allison, Lyons & Del Mar (2004) | x |  | x | x | x | x | x | x | x | x |
| Dubé, Fuller, Rosen, Fagan, O'Donnell (2004) | x |  | x | x | x | x | x | x | x | x |
| Ellis & Campbell (2005) | x |  | x | x | x | x | x | x | x | x |
| O'Day, Killeen, Sutton & Lezzoni (2005) | x |  | x | x | x |  | x | x | x | x |
| Sankar & Jones (2005) | x |  | x | x | x | x | x | x | x | x |
| Ali, Atkin & Neal (2006) |  |  | x | x | x | x |  | x | x | x |
| Cant & Taket (2006) | x |  | x | x | x | x | x | x | x | x |
| Moffat, Cleland, van der Molen & Price (2006) | x |  | x | x | x | x | x | x | x | x |
| Towle, Godolphin & Alexander (2006) | x |  | x | x | x | x | x | x | x | x |
| Vickers, Jolly & Greenfield (2006) | x |  | x | x | x | x | x | x | x | x |
| Abdulhadi, Shafaee, Freudenthal, Östenson & Wahlström (2007) | x |  | x | x | x | x | x | x | x | x |
| Borgsteede, Deliens, Graafland-Riedstra, Francke, van der Wal & Willems (2007) | x |  | x | x | x | x | x | x | x | x |
| Fagerli, Lien & Wandel (2007) | x |  | x | x | x | x | x | x | x | x |
| Kokanovic & Manderson (2007) | x |  | x | x | x | x | x | x | x | x |
| Lowe, Griffiths & Sidhu (2007) | x |  | x | x | x | x | x | x | x | x |
| Mercer, Cawston & Bikker (2007) | x | x | x | x | x | x | x | x | x | x |
| Oliffe & Thorne (2007) | (refer to main study) | (refer to main study) | x | (refer to main study) | x | (refer to main study) | x | x | x | x |
| Julliard, Vivard, Delgado, Cruz, Kabak & Sabers (2008) | x |  | x | x | x | x | x | x | x | x |
| Nguyen, Barg, Armstrong, Holmes & Hornik (2008) | x |  | x | x | x | x | x | x | x | x |
| Smith, Braunack-Mayer, Wittert & Warin (2008) | x |  | x | x | x | x | x | x | x | x |
| Shelley, Sussman, Williams, Segal & Crabtree (2009) | x |  | x | x | x | x | x | x | x | x |
| Wullink, Veldhuijzen, van Schrojenstein, de Valk, Metsemakers & Dinant (2009) | x |  | x | x | x |  | x | x | x | x |
| Matthias, Bair, Nyland, Huffman, Stubbs, Damusb & Kroenke (2010) | x |  | x | x | x |  | x | x | x | x |
| Peek, Odoms-Young, Quinn, Gorawara-Bhat, Wilson & Chin (2010) | x |  | x | x | x |  | x | x | x | x |
| Yorkston, Johnson, Boesflug, Skala & Amtmann (2010) | x |  | x | x | x | x | x | x | x | x |
| Jagosh, Boudreau, Steinert, MacDonald & Ingram (2011) | x |  | x | x | x |  | x | x | x | x |
| Walseth, Abildsnes & Schei (2011) | x | x | x | x | x | x | x | x | x | x |
| Black (2012) | x |  | x | x | x |  | x | x | x | x |
| Burton (2012) | x |  | x |  | x |  | x | x | x | x |
| Dahm (2012) |  |  |  |  |  |  |  |  |  |  |
| Hartley, Sutherland, Brown & Yelland (2012) | x |  | x | x | x | x | x | x | x | x |
| Holmvall, Twohig, Francis & Kelloway (2012) | x |  | x | x | x |  | x | x | x | x |
| Shannon, O'Dougherty & Mehta (2012) | x |  | x | x | x | x | x | x | x | x |
| Weber & Mathews (2012) | x |  | x | x | x |  | x | x | x | x |
| Bergman, Matthias, Coffing & Krebs (2013) |  |  | x | x | x | x | x | x | x | x |
| Claramita, Mubarika, Nugraheni, van Dalen & van der Vleuten (2013) | x |  | x | x | x |  | x | x | x | x |
| Cocksedge, George, Renwick & Chew-Graham (2013) | x | x | x | x | x | x | x | x | x | x |
| Hughes (2013) | x |  | x | x | x | x | x | x | x | x |
| Wilkinson, Dreyfus, Bowen & Bokhour (2013) | x |  | x | x | x |  | x | x | x | x |
| Baumbusch, Phinney, & Baumbusch (2014) | x |  | x | x | x | x | x | x | x | x |
| Bayliss, Riste, Fisher, Wearden, Peters, Lovell, & Chew-Graham (2014) | x |  | x | x | x |  | x | x | x | x |
| Marcinowicz, Pawlikowska, & Oleszczyk (2014) | x |  | x | x | x | x | x | x | x | x |
| Matthias, Krebs, Bergman, Coffing, & Bair (2014) |  | x | x | x | x | x | x | x | x | x |
| Ritholz, Beverly, Brooks, Abrahamson, & Weinger (2014) |  | x | x | x | x | x | x | x | x | x |
| Rose & Harris (2014) | x |  | x | x | x |  | x | x | x | x |
| Esquibel & Borkan (2014) | x |  | x | x | x | x | x | x | x | x |
| Melton, Graff, Holmes, Brown, & Bailey (2014) |  |  | x | x | x |  | x | x | x | x |
| Silver (2015) |  |  | x | x | x | x | x | x | x | x |
